# Implication of miR-612 and miR-1976 in the regulation of *TP53* and *CD40* and their relationship in the response to specific weight-loss diets

**DOI:** 10.1371/journal.pone.0201217

**Published:** 2018-08-08

**Authors:** Marcos Garcia-Lacarte, J. Alfredo Martinez, M. Angeles Zulet, Fermin I. Milagro

**Affiliations:** 1 Department of Nutrition, Food Science and Physiology, University of Navarra, Pamplona, Spain; 2 Center for Nutrition Research, University of Navarra, Pamplona, Spain; 3 CIBERobn, Physiopathology of Obesity and Nutrition, Center of Biomedical Research Network, ISCIII Madrid, Spain; 4 IdiSNA, Navarra's Health Research Institute, Pamplona, Spain; University of Naples Federico II, ITALY

## Abstract

**Background:**

Non-coding RNAs (i.e., miRNAs) play a role in the development of obesity and related comorbidities and the regulation of body weight.

**Objective:**

To identify candidate miRNA biomarkers throughout omics approaches in order to predict the response to specific weight-loss dietary treatments.

**Design:**

Genomic DNA and cDNA isolated from white blood cells of a subset from the RESMENA nutritional intervention study (Low-responders (LR) vs High-responders (HR)) was hybridized in Infinium Human Methylation450 BeadChip and in Illumina Human HT-12 v4 gene expression BeadChips arrays respectively. A bioinformatic prediction of putative target sites of selected miRNAs was performed by applying miRBase algorithms. HEK-293T cells were co-transfected with expression vectors containing the 3’-UTR of candidate genes to validate the binding of miRNAs to its target sites.

**Results:**

134 miRNAs were differentially methylated between HR and LR in the methylation array, whereas 44 miRNAs were differentially expressed between both groups in the expression array. Specifically, miR-1237, miR-1976, miR-642, miR-636, miR-612 and miR-193B were simultaneously hypomethylated and overexpressed in HR. miR-612 and miR-1976 showed greatest differences in methylation and expression levels, respectively. The bioinformatic prediction revealed that *TP53* was a putative target gene of miR-612 and *CD40* of miR-1976. Moreover, *TP53* was downregulated in the expression array when comparing HR vs LR expression levels adjusted by sex, diet, age and baseline weight, and *CD40* showed a statistical trend. Furthermore, gene expression levels of *TP53* and *CD40* in white blood cells, when measured by qPCR, were also downregulated in HR. Finally, miR-612 and miR-1976 potently repressed *TP53* and *CD40* respectively by targeting its 3’-UTR regions.

**Conclusion:**

miR-612 and miR-1976 levels could be prospective biomarkers of response to specific weight-loss diets and might regulate the gene expression of *TP53* and *CD40*.

## Introduction

The rates of obesity incidence have doubled in many countries since 80’s [1]. This growing prevalence and the related disease burdens highlight the need to understand not only the involved process but also to identify and evaluate biomarkers to address this problem. In the last decades, several types of biomarkers have been investigated in relation to their potential application in cardiovascular disease, infections, immunological and genetic disorders, and cancer diagnosis and management [2]. Concerning obesity, different biomarkers of response to different dietary approaches have been identified [3–5].

MicroRNAs (miRNAs) are non-coding RNAs (ncRNAs) of 18–25 nucleotides in length, that bind to a 3’-UTR target mRNA resulting usually in post-transcriptional regulation of gene expression [6] via transcript degradation (when the complementarity is nearly perfect) and/or inhibition of translation or deadenylation (when there are some mismatches in the binding).

On the other hand, miRNAs isolated from white blood cells [7] or directly from the circulation [8] are a good source of biomarkers, and their implication in obesity-related diseases has been well documented [9]. The identification of reliable biomarkers of response to anti-obesity treatments is of crucial importance in order to boost motivation, increase weight loss and maintenance success, and save time and money [10]. In this context, the use of biomarkers that predict the efficacy of weight loss treatments is considered a milestone in the design of precision nutrition strategies against obesity and related comorbidities [11].

In this study, we aimed to identify miRNAs from blood white cells that could be predictive of the outcome of a specific weight-loss intervention. For this purpose, in order to evaluate the interaction between dietary patterns targeting obesity and related transcriptomic biomarkers (miRNAs), we used a miRNAomic approach including methylation and expression microarrays.

## Materials and methods

### Subjects and study protocol

The current study was conducted in a subsample of the RESMENA (Metabolic Syndrome Reduction in Navarra) nutritional intervention trial. In this study, 96 adults with metabolic syndrome underwent two energy-restricted dietary patterns (AHA diet as reference diet, and RESMENA diet as intervention diet) during 8 weeks, both with an energy restriction of -30% of the studied requirements [12]. As no differences were found neither in anthropometric or biochemical variables between groups after the intervention, both dietary groups were merged for further analyses to increase the statistical power of the study, classifying subjects in “high-responders” (HR) when weight loss was ≥8%, and “low responders” (LR) when weight loss was ≤8%, as previously published [4].

The study was performed following the CONSORT 2010 guidelines and properly approved by the Ethics Committee of the University of Navarra (065/2009) and registered at www.clinicaltrials.gov (NTC01087086). All participants provided written informed consent for participation.

### Microarray analyses

Genomic DNA isolated from white blood cells of a subpopulation (31 LR vs 16 HR) of the RESMENA cohort was hybridized in an Infinium HumanMethylation450 BeadChip array (Illumina HM450K). Also, RNA from the same cells (14 LR vs 10 HR) was reverse transcribed and hybridized in an Illumina Human HT-12 v4 gene expression BeadChip array. Microarray data were analysed using Limma package in R [13]. Corrections for multiple comparisons were carried out in both microarrays (expression and methylation) by using the Benjamini-Hochberg procedure.

### Bioinformatic study

A bioinformatic study of putative target sites of selected miRNAs was performed by applying miRBase algorithms (www.mirbase.org). For each miRNA, miRBase provides references in literature, genomic coordinates and links to databases of predicted and validated miRNA target sites such as DIANA-microT, microRNA.org, miRDB, RNA22, TargetMiner and TargetScan [14].

### MassArray Epityper validation

In order to validate the results of the methylation microarray, miR-612 methylation levels of 47 subjects selected from the subpopulation sample were analyzed by MassArray EpiTyp0065r (Sequenom, San Diego, CA, USA) after designing specific primers encompassing 6 CpGs sites (F: GTTTTATGGTAGTGGGAAGGGATTT; R: AATAAAACCCAAACAACAAACAATC). This method has been previously applied to validate methylation microarray data [15].

### Luciferase reporter constructs

To verify if selected miRNAs regulate the 3’-UTR of the two predicted target genes, expression vectors containing the 3’-UTR region of each gene provided by the bioinformatic prediction were designed. To amplify the 3’-UTR region of *TP53* and *CD40* genes, specific primers incorporating *NheI* and *XbaI* restriction enzymes sites were designed. The PCR products were subsequently cloned downstream of the firefly luciferase (*luc*) gene in the pmiR-GLO Dual-Luciferase miRNA Target Expression Vector (Promega, Madison, WI, USA) (S1 Fig). Primer sequences are shown in Table 1.

**Table 1:** Primer sequences used to amplify the 3’-UTR region of target genes and the amplicon length.

*CD40*-F	5’-TTAGCTAGCACTTTACATGGATGCCAACC-3’	766 bp
*CD40*-R	5’-TTATCTAGACACCACTCTTCGAGCTGT-3’	
*TP53*-F	5’-TTAGCTAGCGCCAAACCCTGTCTGACAA-3’	1010 bp
*TP53*-R	5’-TTATCTAGAAACCCAGGTATCCTGCCA-3’	

F: Forward. R: Reverse. Underlined: *NheI* and *XbaI* target sites

### Cell culture

Human HEK-293T cells were purchased from the ATCC and maintained in Dulbecco’s Modified Eagle’s Medium (DMEM) supplemented with 10% fetal bovine serum (FBS) and 100 U/ml penicillin-streptomycin at 37ºC in a 5% carbon dioxide humidified atmosphere.

### Dual-luciferase reporter assays

To assess miRNA-target interactions, HEK-293T cells were seeded in 96-well plates at a density of 20000 cells per well. After 8 h, cells were transiently co-transfected with either 0.25 μg of empty pmiR-GLO, pmiR-GLO-*TP53*-3’-UTR, or pmiR-GLO-*CD40*-3’-UTR, and 7.5 pmol of miR-612 and miR-1976 mimics using Lipofectamine 2000 (Invitrogen, Carlsbad, CA, USA) according to manufacturer’s protocol. Firefly and *Renilla* luciferase activities were evaluated 24 h after co-transfection using a Dual-Luciferase Reporter Assay System (Promega, Madison, WI, USA). Firefly luciferase activity was normalized using *Renilla* luciferase activity. Determinations were carried out in three independent experiments, each assayed in triplicate.

### RNA isolation and quantitative real-time PCR

RNA from white blood cells was extracted using TRIzol reagent (Life Technologies, Carlsbad, CA, USA) according to manufacturer’s protocol. cDNA was synthesized from 0.5 ug of total RNA using random primers and MultiScribe^TM^ MuLV reverse transcriptase (Applied Biosystems, Foster City, CA, USA). For mature miR-1976, 20 ng of RNA were reverse transcribed by using a Taqman MicroRNA RT kit (Applied Biosystems) and miRNA-specific primer sets supplied by the manufacturer. Quantitative real time PCR (qPCR) was performed with the ABI prism 7900HT Sequence Detection System (Applied Biosystems) using Taqman probes. Both mRNAs and miRNAs relative expression was calculated with the 2^-ΔΔ*CT*^ method and normalized using *GAPDH* and U48 mature miRNA, respectively.

### Statistical analysis

Differences between groups were calculated using the Student’s t-test or an ANCOVA test when indicated. Data are presented as mean ± SEM. p-values less than 0.05 were defined as statistically significant. Volcano plots were created by plotting the negative log_10_ of the p-value (y axis) and the mean differences between groups for each variable (x axis). An effect size of ±1.5% in the methylation differences and an effect size of ±1% in the expression differences were considered of interest. Statistical analyses and graphics were performed using SPSS 15.0 software (SPSS Inc., Chicago, IL, USA) and GraphPad Prism version 6.0C (La Jolla, CA, USA).

## Results

### miR-612 and miR-1976 are hypomethylated and overexpressed in HR

**Fig 1:**
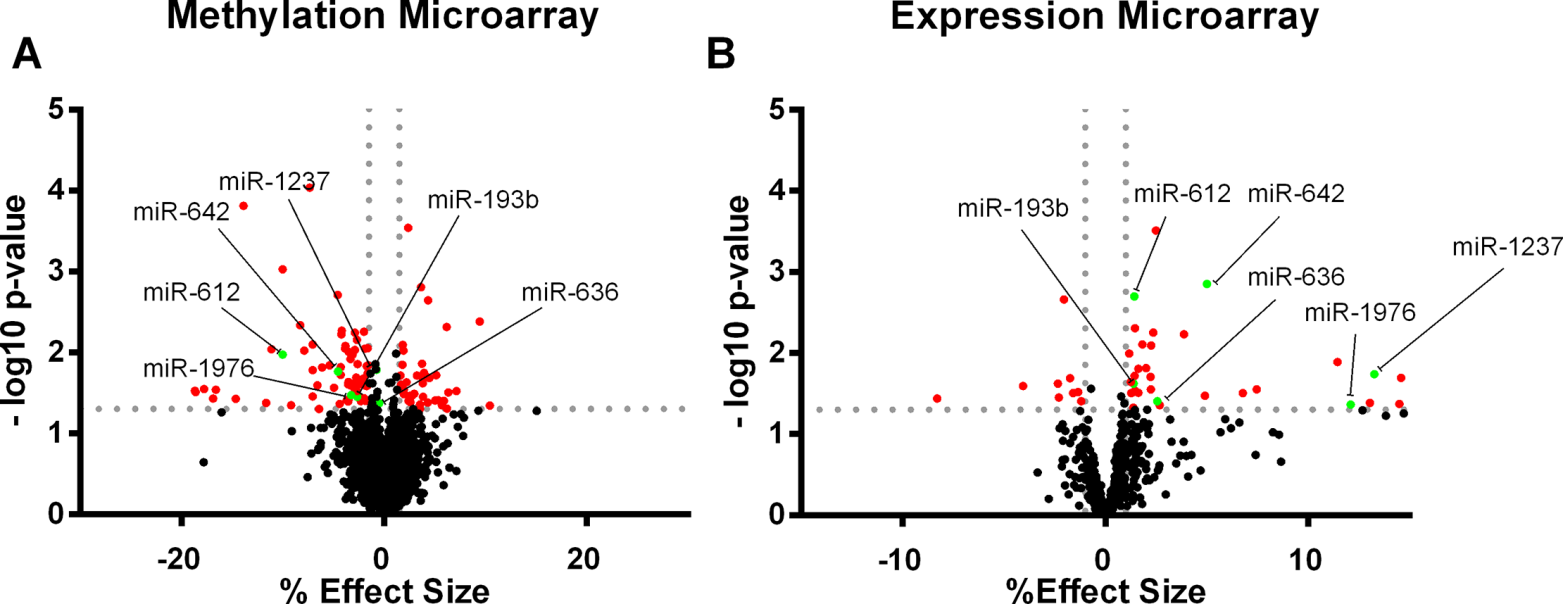
Identification of miR-612 and miR-1976 in the methylation and expression microarrays. A) Volcano plot of miRNAs differentially methylated between HR and LR. B) Volcano plot of miRNAs differentially expressed between HR and LR.

When analyzing array data, 134 miRNAs differentially methylated (87 hypomethylated and 47 hypermethylated), and 44 miRNAs differentially expressed (10 downregulated and 34 upregulated) were identified when comparing HR and LR (S1 and S2 Tables). From them, miR-1237, miR-1976, miR-642, miR-636, miR-612 and miR-193B were simultaneously hypomethylated and overexpressed in HR (Fig 1A and 1B). miR-612 showed significant difference in expression levels (1.43% effect size; p = 0.019) and the greatest difference in methylation levels (10% effect size; p = 0.003). Likewise, miR-1976 showed also significant difference in methylation levels (3.24% effect size; p = 0.041) and greatest differences in expression levels (12% effect size; p = 0.043). Although nominal statistically differences in miR-612 and miR-1976 between HR and LR were found, they disappeared after correction for multiple comparisons (Benjamini-Hochberg). Nevertheless, as these miRNAs showed notable differences in both methylation and expression microarrays, they were selected for further evaluation.

### *TP53* is a putative target gene of miR-612

**Fig 2:**
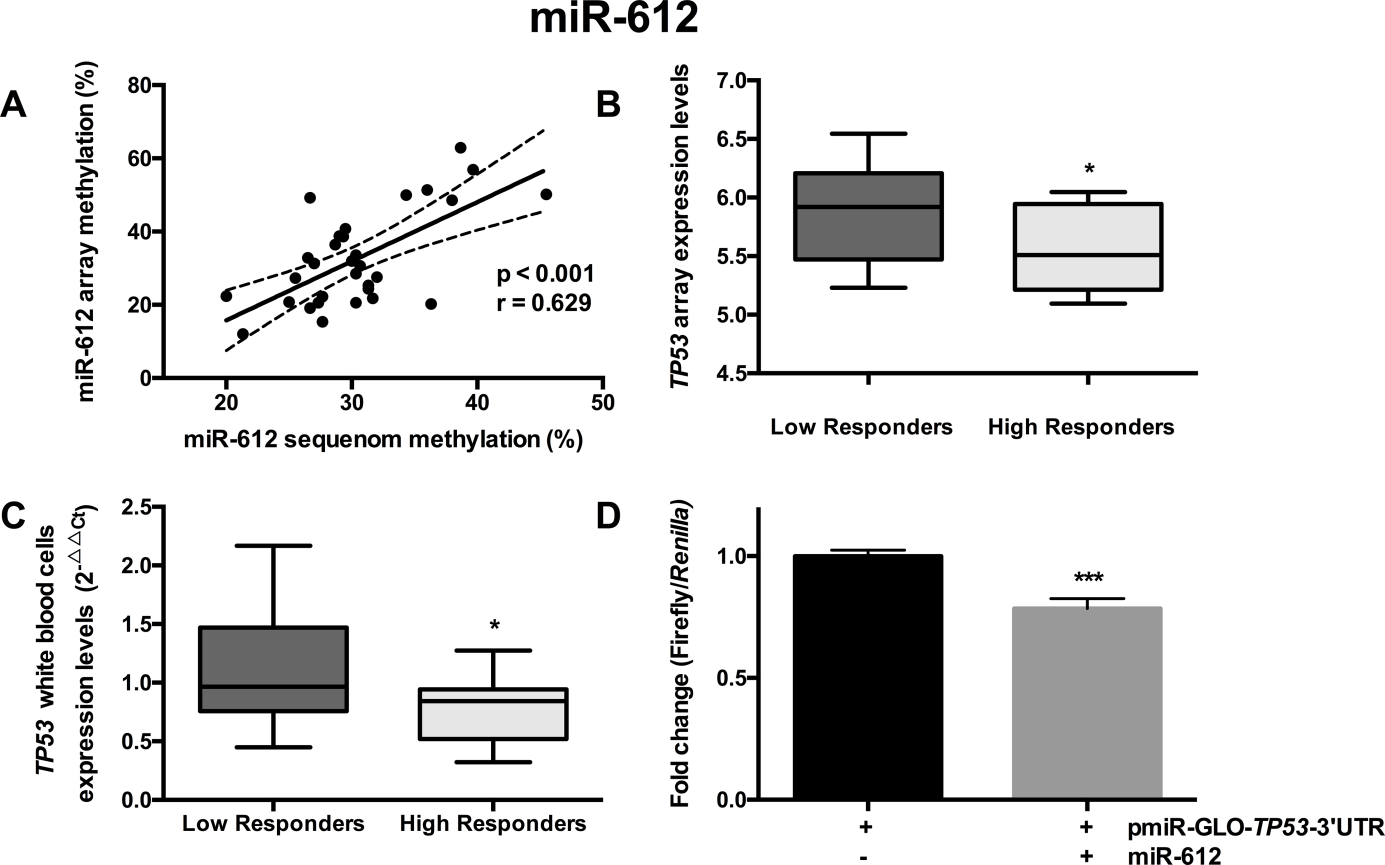
Validation of miR-612 and its target gene *TP53*. A) Correlation between miR-612 methylation levels by microarray and EpiTyper. B) Gene expression of *TP53* in HR and LR in the microarray. C) Validation of *TP53* expression profile in HR and LR white blood cells by qPCR, adjusted for age, sex, diet and baseline body weight. * p < 0.05 from ANCOVA test. D) Luciferase activity assay of pmiR-GLO-*TP53*-3’-UTR after co-transfection with miR-612. Normalized luciferase activity is presented as the mean ± SEM of three separate triplicate experiments. *** p < 0.001 from Student t-test.

To select putative miRNAs implicated in the response to the diet, a bioinformatic study using miRBase algorithms assigning p-values to putative target binding sites of those miRNAs simultaneously hypomethylated and overexpressed in arrays was carried out. We then focused on obesity-related genes and filtered the target sites of each miRNA. We noted that *TP53* was predicted to be regulated by miR-612. EpiTyper analysis of the DNA methylation levels of miR-612 in white blood cells showed a positive correlation (p < 0.001) with respect to the DNA methylation levels measured by microarray (Fig 2A). Moreover, *TP53* was downregulated in the expression array (p = 0.024) when comparing HR vs LR expression levels adjusted by sex, diet, age and baseline weight (Fig 2B). Furthermore, *TP53* levels were also significantly lower in HR than in LR when measured by qPCR (p = 0.04), supporting the idea that miR-612 might affect *TP53* gene expression (Fig 2C). Finally, cells co-transfected with the pmiR-GLO-*TP53*-3’-UTR vector and miR-612 showed significantly lower firefly/*Renilla* activity (p < 0.001) than controls transfected only with the pmiR-GLO-*TP53*-3’-UTR vector (Fig 2D), confirming that *TP53* is a target gene of miR-612.

### *CD40* is a putative target gene of miR-1976

**Fig 3:**
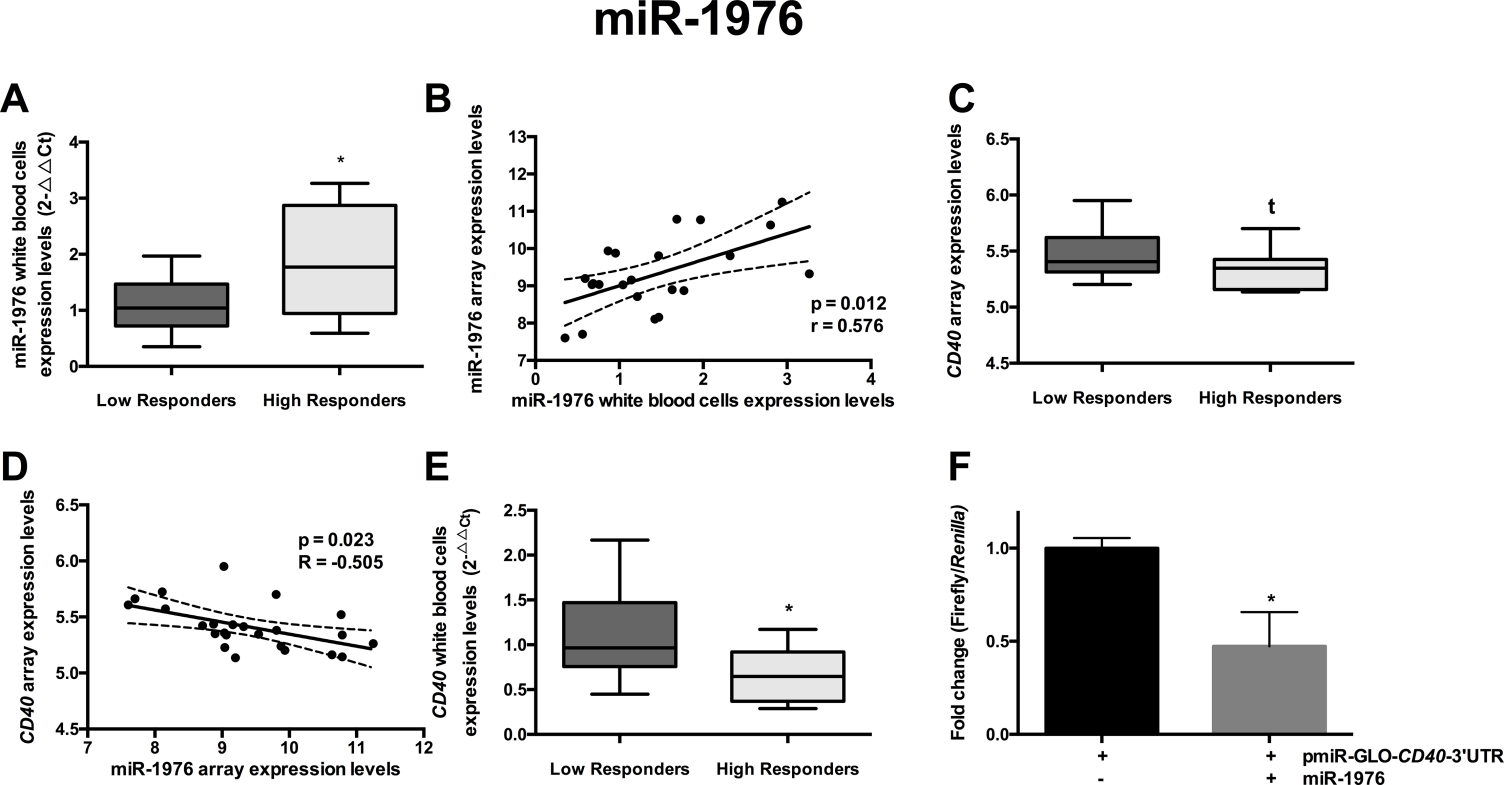
Validation of miR-1976 and its target gene *CD40*. A) miR-612 expression levels by qPCR in HR and LR, adjusted for age, sex, diet and baseline body weight. * p < 0.05 from ANCOVA test. B) Pearson’s correlation between miR-1976 expression levels in the microarray and by qPCR, adjusted by sex, age, baseline body weight and diet. C) Gene expression of *CD40* in HR and LR in the microarray. t p = 0.069. D) Pearson’s correlations between miR-1976 expression levels and its target gene (*CD40*) in the microarray, adjusted by sex, age, baseline body weight and diet. E) Validation of *CD40* expression profile in HR and LR white blood cells by qPCR adjusted for age, sex, diet and baseline body weight. * p < 0.05 from ANCOVA test. F) Luciferase activity assays of pmiR-GLO-*CD40*-3’-UTR after co-transfection with miR-1976. Normalized luciferase activity is presented as the mean ± SEM of three separate triplicate experiments. * p < 0.05 from Student t-test.

Similarly to miR-612, we carried out the same approach to identify putative obesity-related target genes of miR-1976. According to miRBase, *CD40* could be regulated by miR-1976. First, we found that miR-1976 expression profile in white blood cells was statistically different between HR and LR (p = 0.019) and also showed a positive correlation (p = 0.012) with miR-1976 expression levels measured by microarray (Fig 3A–3B). *CD40* expression level showed a trend toward significance (p = 0.069) when comparing HR vs LR adjusted by sex, diet, age and baseline weight (Fig 3C). Interestingly, gene expression levels of *CD40* were also significant negatively correlated (p = 0.023; R = -0.505) with miR-1976 expression profile (Fig 3D). Furthermore, *CD40* expression in white blood cells, when measured by qPCR, were significantly lower in HR than in LR (p = 0.02) (Fig 3E), suggesting an interaction between miR-1976 and *CD40*. Similarly, cells co-transfected with the pmiR-GLO-*CD40*-3’-UTR construct and miR-1976 showed also a significantly reduction in firefly/*Renilla* activity (p = 0.014) than controls transfected only with the expression vector (Fig 3F), confirming that *CD40* is a target gene of miR-1976.

## Discussion

In this study, a miRNAomic approach was performed in order to find transcriptomic biomarkers (especially miRNAs) associated to the response to specific weight loss diets. It is well established that miRNAs can regulate the expression of genes by binding to its target sites, usually resulting in degradation or translation inhibition [6]. miRNAs have been implicated in many development and diseases processes [16], including obesity and associated comorbidities [17]. For example, in type 2 diabetes, a well-known obesity-related disease, several miRNAs are down or upregulated [18]. miR-103 and miR-107 have been reported to contribute to adipose growth by accelerating adipocyte differentiation, and both are upregulated in obese individuals [19]. Moreover, several miRNAs have been defined as important modulators in human obesity-related inflammation [20] or white adipose tissue inflammation [21], adipogenesis and adipose tissue signaling [22]. Additionally, changes in miRNA levels in plasma, serum, urine and other fluids have been associated with different diseases such as prostate cancer [23], bladder cancer [24] or cell carcinoma [25]. Thus, the identification of circulating miRNAs could serve as useful clinical biomarkers of diagnosis and prognosis of several diseases [26].

The present study has demonstrated that miR-612 and miR-1976 bind to *TP53* and *CD40* respectively and regulate their expression. *TP53* gene encodes p53 protein, a tumor suppressor whose deficiency enhances the initiation and/or progression of cancer [27]. Noticeably, Minamino et al. found that the expression of proinflammatory cytokines in mice decreased and insulin resistance improved after inhibition of p53 in adipose tissue, suggesting an important role of p53 in the regulation of obesity-related inflammation and insulin resistance [28]. Furthermore, they also evidenced that adipose tissue from subjects with diabetes showed higher levels of p53 protein compared with tissue from nondiabetic subjects, and that the expression of inflammatory cytokines was also significantly increased in adipose tissue.

Moreover, *ob/ob* mice show higher levels of p53 than wild type mice, and the disruption of p53 in *ob/ob* mice restores the expression of lipogenic enzymes [29]. Conversely, Molchadsky *et al*. showed that p53 may exert either a positive or negative effect according to the adipogenic differentiation program [30]. In our study, those subjects who responded better to the diet had lower expression of *TP53* than LR. It can be speculated that LR had higher inflammatory state than HR, and that this inflammation could trigger an activation of *TP53*.

In white blood cells, *TP53* is downregulated in obese subjects with type 2 diabetes after bariatric surgery, suggesting that *TP53* is upregulated in white blood cells of obese subjects [31]. Taking all these data together, our findings of decrease whole blood *TP53* mRNA in HR are consistent with these studies.

Several *TP53*-directed miRNAs have been experimentally and *in silico* identified. Some of these miRNAs that target *TP53* are miR-125b, miR-504, miR-1285, miR-92, miR-141, miR-380-5p, miR-15a, miR-16, miR-25, miR-30d, miR-200a, miR-453, miR-98, miR-19b, miR-518c and miR-638 [32]. Interestingly, miR-1285 has the same seed sequence as miR-612. In a previous study, Tian *et al*. tried to validate the binding of miR-612 to *TP53* and found that the luciferase activity of the p-LUC-p53-3’-UTR reporter did not change when transiently transfected with miR-612 [33]. However, in the present study, our data show that miR-612 binds to the 3’-UTR of *TP53* and that there exists a negative relationship between miR-612 levels and *TP53* expression (in blood and in the microarray), indicating that miR-612 could regulate *TP53* expression.

On the other hand, CD40 is a surface glycoprotein expressed in hematopoietic and nonhematopoietic cells, and has an important role in the ability to stimulate adaptive immunity [34]. Concerning obesity, *CD40* is highly expressed in leukocytes, adipocytes and the stromal cells of adipose tissue [35], and is involved in the regulation of adipose tissue metabolism [36]. Furthermore, soluble CD40L levels have been positively correlated with obesity and metabolic syndrome [37,38], and studies in rodents have shown that vascular inflammation and atherosclerosis could be prevented by CD40 deficiency [39,40]. There exists evidence that *CD40* could be regulated by miRNAs [41,42], even though to date there are no articles showing a miRNA-regulation of *CD40* in obese subjects.

To our knowledge, this is the first study to explore the relationships between miR-612 and miR-1976 and *TP53* and *CD40*, respectively, and was able to connect the expression of these miRNAs and genes with the response to a dietary intervention in obese subjects. However, further studies are needed to better understand the complex regulation of these miRNAs on their target genes. Transcriptomic biomarkers of the response to specific dietary strategies are a first step towards the personalization of weight-loss treatment, being miRNAs particularly relevant for this purpose.

## Supporting information

S1 Fig miR-612 and miR-1976 regulate the 3’-UTR region of *TP53* and *CD40*, respectively. A) Location of putative target sites for miR-612 and miR-1976 in the 3’-UTR of *TP53* and *CD40* predicted by TargetScan. B) miR-GLO Dual-Luciferase miRNA Target Expression Vector used to create the 3’-UTR expression vectors cloning the PCR product into the MCS. MCS: Multiple Cloning Site.
(PDF)

S1 Table Significantly differentiated methylated miRNAs between HR and LR. In bold style, those miRNAs that were above the selected threshold of ±1.5%.
(DOCX)

S2 Table Significantly differentiated expressed miRNAs between HR and LR. In bold style, those miRNAs that were above the selected threshold of ±1%.
(DOCX)
