# Supplementary material for: Implication of miR-612 and miR-1976 in the regulation of TP53 and CD40 and their relationship in the response to specific weight-loss diets
Source: PLoS One. 2018 Aug 8;13(8):e0201217. doi: 10.1371/journal.pone.0201217 (PMC6082528; doi:10.1371/journal.pone.0201217)
Supplement: S1 Fig — A) Location of putative target sites for miR-612 and miR-1976 in the 3’-UTR of TP53 and CD40 predicted by TargetScan. B) miR-GLO Dual-Luciferase miRNA Target Expression Vector used to create the 3’-UTR expression vectors cloning the PCR product into the MCS. MCS: Multiple Cloning Site. (PDF) [file pone.0201217.s001.pdf]

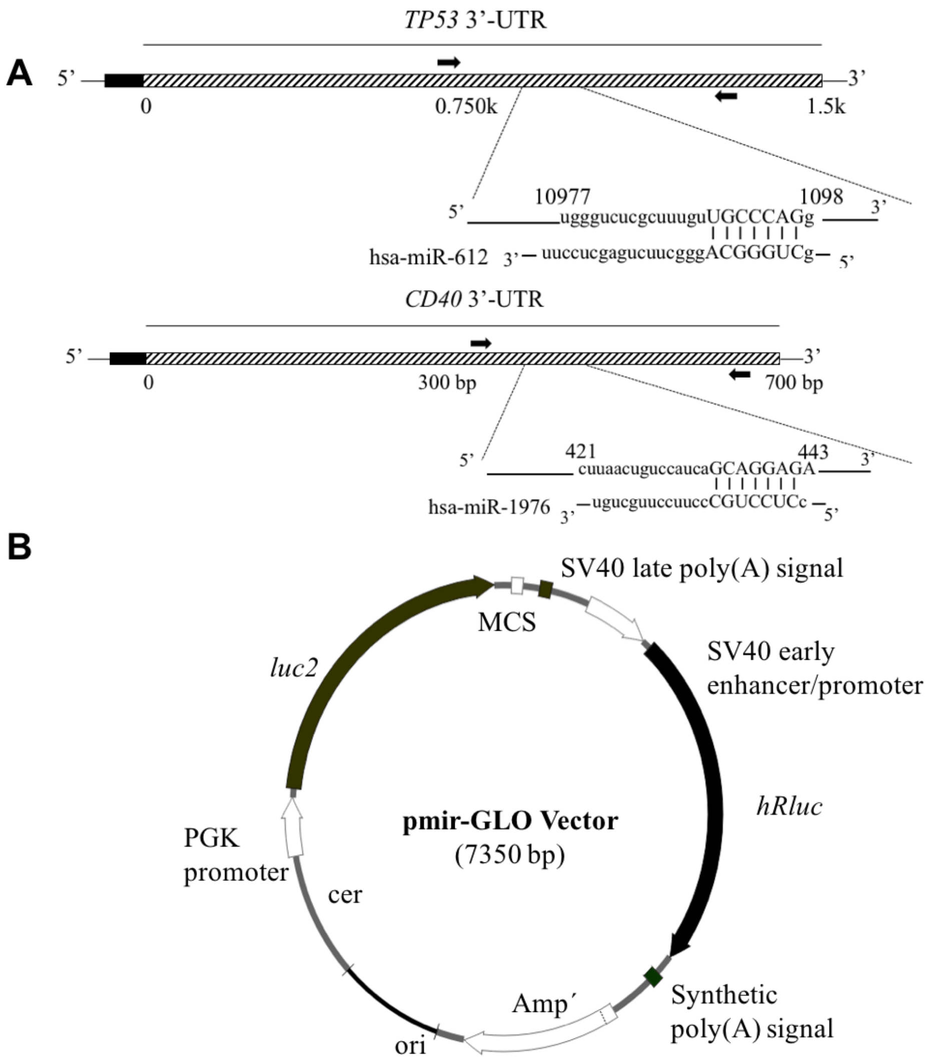

S1 Fig. miR-612 and miR-1976 regulate the 3'-UTR region of *TP53* and *CD40*, respectively. A) Location of putative target sites for miR-612 and miR-1976 in the 3'-UTR of *TP53* and *CD40* predicted by TargetScan. B) miR-GLO Dual-Luciferase miRNA Target Expression Vector used to create the 3'-UTR expression vectors cloning the PCR product into the MCS. MCS: Multiple Cloning Site
