# Supplementary material for: Implication of miR-612 and miR-1976 in the regulation of TP53 and CD40 and their relationship in the response to specific weight-loss diets
Source: PLoS One. 2018 Aug 8;13(8):e0201217. doi: 10.1371/journal.pone.0201217 (PMC6082528; doi:10.1371/journal.pone.0201217)
Supplement: S1 Table — In bold style, those miRNAs that were above the selected threshold of ±1.5%. (DOCX) [file pone.0201217.s002.docx]

| **S1 Table. Significantly differentiated methylated miRNAs between HR and LR.** In bold style, those miRNAs that were above the selected threshold of ±1.5 %. | | | |
| --- | --- | --- | --- |
| Name | % Effect Size | "-logP-value" | p-value |
| **MIR505** | -7.356 | 4.033 | 0.0001 |
| **MIR1284** | -13.876 | 3.808 | 0.0002 |
| **MIR2116** | 2.371 | 3.540 | 0.0003 |
| **MIR548N** | -10.024 | 3.026 | 0.0009 |
| **MIRLET7G** | 3.666 | 2.807 | 0.0016 |
| **MIR141** | -4.585 | 2.710 | 0.0020 |
| **MIR548H4** | 4.360 | 2.644 | 0.0023 |
| **MIR548N** | 9.442 | 2.380 | 0.0042 |
| **MIR487B;MIR539** | -8.283 | 2.333 | 0.0046 |
| **MIR1256** | 6.190 | 2.315 | 0.0048 |
| **MIR657;MIR338** | -4.195 | 2.267 | 0.0054 |
| **MIR130A** | -1.980 | 2.254 | 0.0056 |
| **MIRLET7A3;MIRLET7B** | -2.880 | 2.242 | 0.0057 |
| **MIRLET7B** | -4.224 | 2.221 | 0.0060 |
| **MIR525;MIR523** | -2.688 | 2.157 | 0.0070 |
| **MIR647** | -7.063 | 2.097 | 0.0080 |
| **MIR220B** | 1.865 | 2.094 | 0.0081 |
| **MIR130A** | -3.808 | 2.082 | 0.0083 |
| **MIR375** | -1.633 | 2.052 | 0.0089 |
| **MIR935** | -3.842 | 2.050 | 0.0089 |
| **MIR106B** | -1.893 | 2.050 | 0.0089 |
| **MIR487B;MIR539** | -11.121 | 2.039 | 0.0091 |
| **MIR182** | -2.899 | 2.032 | 0.0093 |
| **MIR301A** | 1.913 | 2.023 | 0.0095 |
| **MIR1208** | -7.880 | 2.021 | 0.0095 |
| **MIR130B;MIR301B** | -3.497 | 2.005 | 0.0099 |
| MIR199A1 | 1.175 | 1.987 | 0.0103 |
| **MIR200A;MIR429** | -3.108 | 1.974 | 0.0106 |
| **MIR612** | -10.032 | 1.971 | 0.0107 |
| **MIR671** | -3.322 | 1.919 | 0.0120 |
| **MIR654;MIR376B;MIR376A2;MIR300;MIR376A1** | 3.755 | 1.861 | 0.0138 |
| MIR548J | -0.845 | 1.858 | 0.0139 |
| **MIR1537** | -2.602 | 1.856 | 0.0139 |
| **MIR512-1;MIR512-2** | 1.826 | 1.846 | 0.0142 |
| **MIR662** | -5.388 | 1.841 | 0.0144 |
| **MIR628** | -1.587 | 1.841 | 0.0144 |
| **MIRLET7C** | -1.717 | 1.832 | 0.0147 |
| **MIR637** | -4.269 | 1.819 | 0.0152 |
| **MIR339** | -6.132 | 1.816 | 0.0153 |
| MIR1250 | -0.822 | 1.794 | 0.0161 |
| MIR1237 | -0.759 | 1.786 | 0.0164 |
| **MIR1304** | -7.064 | 1.780 | 0.0166 |
| **MIR642** | -4.535 | 1.765 | 0.0172 |
| **MIR138-1** | 3.953 | 1.750 | 0.0178 |
| **MIR548H3** | -1.607 | 1.745 | 0.0180 |
| MIR1178 | -1.343 | 1.739 | 0.0182 |
| **MIR345** | -4.291 | 1.725 | 0.0189 |
| **MIR200B** | 5.144 | 1.717 | 0.0192 |
| **MIR621** | 3.250 | 1.715 | 0.0193 |
| **MIR220B** | 1.614 | 1.713 | 0.0194 |
| MIR548F1 | 1.225 | 1.698 | 0.0201 |
| **MIR520A** | 4.313 | 1.691 | 0.0204 |
| **MIR431** | -2.735 | 1.688 | 0.0205 |
| **MIR21** | 4.475 | 1.684 | 0.0207 |
| **MIR631** | -2.057 | 1.679 | 0.0210 |
| **MIR548F5** | 3.852 | 1.665 | 0.0216 |
| **MIRLET7I** | -2.746 | 1.650 | 0.0224 |
| **MIR1275** | -3.527 | 1.637 | 0.0231 |
| **MIR34A** | -2.566 | 1.634 | 0.0233 |
| **MIR205** | 2.230 | 1.633 | 0.0233 |
| MIR548G | 0.712 | 1.626 | 0.0237 |
| MIR423 | 0.492 | 1.624 | 0.0238 |
| MIR888 | -1.406 | 1.620 | 0.0240 |
| **MIR516A2;MIR519A2** | 3.825 | 1.619 | 0.0241 |
| MIR432;MIR136 | -1.165 | 1.617 | 0.0241 |
| **MIR675** | -2.435 | 1.615 | 0.0243 |
| **MIR124-2** | -2.390 | 1.612 | 0.0244 |
| **MIR520A** | -3.536 | 1.606 | 0.0248 |
| **MIR548H4** | 1.689 | 1.601 | 0.0251 |
| **MIR548H3** | -1.595 | 1.597 | 0.0253 |
| **MIR199A2** | -2.010 | 1.596 | 0.0253 |
| **MIR199A1** | -3.325 | 1.595 | 0.0254 |
| **MIR196A1** | -6.568 | 1.594 | 0.0255 |
| **MIR483** | -2.164 | 1.586 | 0.0259 |
| **MIR758;MIR1197;MIR329-1;MIR323** | -1.710 | 1.584 | 0.0261 |
| **MIR637** | -3.042 | 1.576 | 0.0266 |
| **MIR301B;MIR130B** | -4.952 | 1.564 | 0.0273 |
| **MIR886** | -17.790 | 1.546 | 0.0284 |
| **MIR885** | -2.891 | 1.543 | 0.0287 |
| **MIR340** | 1.939 | 1.541 | 0.0287 |
| **MIR886** | -16.612 | 1.541 | 0.0288 |
| MIR758;MIR1197;MIR329-1;MIR323 | 1.290 | 1.538 | 0.0290 |
| **MIR886** | -18.667 | 1.525 | 0.0298 |
| **MIR147** | -2.688 | 1.523 | 0.0300 |
| **MIR1204** | 7.158 | 1.521 | 0.0301 |
| MIR609 | -0.655 | 1.516 | 0.0305 |
| **MIR886** | -18.632 | 1.510 | 0.0309 |
| **MIR921** | 6.336 | 1.505 | 0.0313 |
| **MIR1236** | 2.969 | 1.491 | 0.0323 |
| **MIR106B;MIR93** | 1.869 | 1.485 | 0.0327 |
| **MIR551B** | 1.962 | 1.484 | 0.0328 |
| **MIR1976** | -3.236 | 1.474 | 0.0336 |
| **MIR548A2** | 2.776 | 1.468 | 0.0340 |
| **MIR30E** | 2.582 | 1.463 | 0.0344 |
| **MIR548G** | -7.054 | 1.457 | 0.0349 |
| **MIR635** | -3.395 | 1.452 | 0.0353 |
| **MIR193B** | -2.664 | 1.451 | 0.0354 |
| MIR638 | -0.760 | 1.451 | 0.0354 |
| **MIR1256** | 3.851 | 1.451 | 0.0354 |
| **MIR662** | -2.420 | 1.450 | 0.0354 |
| MIR1280 | -1.182 | 1.435 | 0.0367 |
| **MIR101-1** | -1.989 | 1.434 | 0.0368 |
| **MIR886** | -16.875 | 1.430 | 0.0372 |
| **MIR886** | -14.634 | 1.428 | 0.0374 |
| **MIR200B;MIR200A** | -3.688 | 1.411 | 0.0388 |
| **MIR494** | -3.509 | 1.410 | 0.0389 |
| MIR548N | 0.969 | 1.410 | 0.0389 |
| **MIR525;MIR523** | 5.365 | 1.409 | 0.0390 |
| **MIR548Q** | 5.065 | 1.408 | 0.0391 |
| **MIR758;MIR329-2;MIR329-1;MIR323** | -3.600 | 1.406 | 0.0393 |
| **MIR548D2** | 2.402 | 1.404 | 0.0394 |
| **MIR575** | -1.830 | 1.402 | 0.0396 |
| **MIR2114** | 5.919 | 1.401 | 0.0397 |
| **MIR2276** | -2.268 | 1.399 | 0.0399 |
| **MIR1469** | -3.557 | 1.392 | 0.0406 |
| **MIR1236** | 2.613 | 1.387 | 0.0410 |
| **MIR302D;MIR302A;MIR302B;MIR302C;MIR367** | 4.318 | 1.380 | 0.0417 |
| **MIR496;MIR154** | -2.363 | 1.378 | 0.0419 |
| **MIR886** | -11.635 | 1.376 | 0.0421 |
| MIR127 | -1.182 | 1.374 | 0.0422 |
| MIR636 | -0.469 | 1.373 | 0.0423 |
| **MIR145** | -4.373 | 1.365 | 0.0431 |
| MIR1205 | 0.906 | 1.362 | 0.0435 |
| **MIR92A1;MIR17HG;MIR19B1** | 3.541 | 1.355 | 0.0441 |
| **MIR548C** | 5.725 | 1.347 | 0.0450 |
| **MIR886** | -9.188 | 1.346 | 0.0450 |
| **MIR592** | 10.436 | 1.344 | 0.0453 |
| MIR639 | -1.236 | 1.336 | 0.0461 |
| MIR1260 | 0.875 | 1.318 | 0.0481 |
| MIR611 | -0.937 | 1.313 | 0.0486 |
| MIR135B | -1.269 | 1.308 | 0.0492 |
| **MIR138-2** | 6.203 | 1.305 | 0.0496 |
| **MIR365-2** | 3.558 | 1.303 | 0.0498 |
| **MIR1203** | -6.413 | 1.301 | 0.0500 |
