# Supplementary material for: Implication of miR-612 and miR-1976 in the regulation of TP53 and CD40 and their relationship in the response to specific weight-loss diets
Source: PLoS One. 2018 Aug 8;13(8):e0201217. doi: 10.1371/journal.pone.0201217 (PMC6082528; doi:10.1371/journal.pone.0201217)
Supplement: S2 Table — In bold style, those miRNAs that were above the selected threshold of ±1%. (DOCX) [file pone.0201217.s003.docx]

| **S2 Table. Significantly differentiated expressed miRNAs between HR and LR.** In bold style, those miRNAs that were above the selected threshold of ±1%. | | | |
| --- | --- | --- | --- |
| Name | % Effect Size | "-logP-value" | p-value |
| **MIR623** | 2.491 | 3.510 | 0.0003 |
| **MIR642** | 5.007 | 2.853 | 0.0014 |
| **MIR612** | 1.427 | 2.699 | 0.0020 |
| **MIRLET7C** | -2.045 | 2.660 | 0.0022 |
| **MIR668** | 1.459 | 2.305 | 0.0050 |
| **MIR548K** | 2.351 | 2.253 | 0.0056 |
| **MIR542** | 3.872 | 2.232 | 0.0059 |
| **MIR497** | 1.819 | 2.106 | 0.0078 |
| **MIR1200** | 2.261 | 2.094 | 0.0081 |
| **MIR539** | 1.181 | 1.996 | 0.0101 |
| **MIR2116** | 11.447 | 1.891 | 0.0129 |
| **MIR890** | 2.009 | 1.816 | 0.0153 |
| **MIR1257** | 1.626 | 1.805 | 0.0157 |
| **MIR1237** | 13.262 | 1.739 | 0.0182 |
| **MIR449B** | 1.448 | 1.719 | 0.0191 |
| **MIR892A** | 2.232 | 1.707 | 0.0196 |
| **MIR324** | 14.582 | 1.692 | 0.0203 |
| **MIR576** | -1.745 | 1.689 | 0.0205 |
| **MIR196B** | 1.264 | 1.645 | 0.0227 |
| **MIR193B** | 1.391 | 1.623 | 0.0238 |
| **MIR128-1** | -2.345 | 1.622 | 0.0239 |
| **MIR342** | -4.060 | 1.596 | 0.0254 |
| MIR152 | -0.716 | 1.561 | 0.0275 |
| **MIR1185-1** | 7.457 | 1.553 | 0.0280 |
| **MIR548B** | 2.249 | 1.552 | 0.0280 |
| **MIR761** | 1.452 | 1.535 | 0.0292 |
| **MIR543** | -1.344 | 1.517 | 0.0304 |
| **MIR590** | 1.313 | 1.510 | 0.0309 |
| **MIR495** | 1.621 | 1.509 | 0.0310 |
| **MIR548I2** | -1.586 | 1.508 | 0.0310 |
| **MIR943** | 6.773 | 1.507 | 0.0311 |
| **MIR509-2** | 1.280 | 1.505 | 0.0313 |
| **MIR1238** | 4.905 | 1.474 | 0.0336 |
| MIR765 | 0.779 | 1.465 | 0.0343 |
| **MIR571** | -2.291 | 1.451 | 0.0354 |
| **MIR155HG** | -8.295 | 1.438 | 0.0365 |
| **MIR636** | 2.557 | 1.407 | 0.0392 |
| **MIR147** | -1.191 | 1.405 | 0.0393 |
| **MIR939** | 13.048 | 1.386 | 0.0411 |
| MIR520H | 0.936 | 1.377 | 0.0420 |
| **MIR877** | 14.486 | 1.373 | 0.0424 |
| **MIR1976** | 12.089 | 1.367 | 0.0430 |
| **MIR1236** | 2.670 | 1.355 | 0.0442 |
| **MIR617** | 1.372 | 1.326 | 0.0472 |
